# Acid-base transport in pancreas—new challenges

**DOI:** 10.3389/fphys.2013.00380

**Published:** 2013-12-20

**Authors:** Ivana Novak, Kristian A. Haanes, Jing Wang

**Affiliations:** Department of Biology, University of CopenhagenCopenhagen, Denmark

**Keywords:** bicarbonate transport, proton transport, H^+^-K^+^-ATPase, K_Ca_3.1, IK, TMEM16A, ANO1, pancreatic duct

## Abstract

Along the gastrointestinal tract a number of epithelia contribute with acid or basic secretions in order to aid digestive processes. The stomach and pancreas are the most extreme examples of acid (H^+^) and base (HCO^−^_3_) transporters, respectively. Nevertheless, they share the same challenges of transporting acid and bases across epithelia and effectively regulating their intracellular pH. In this review, we will make use of comparative physiology to enlighten the cellular mechanisms of pancreatic HCO^−^_3_ and fluid secretion, which is still challenging physiologists. Some of the novel transporters to consider in pancreas are the proton pumps (H^+^-K^+^-ATPases), as well as the calcium-activated K^+^ and Cl^−^ channels, such as K_Ca_3.1 and TMEM16A/ANO1. Local regulators, such as purinergic signaling, fine-tune, and coordinate pancreatic secretion. Lastly, we speculate whether dys-regulation of acid-base transport contributes to pancreatic diseases including cystic fibrosis, pancreatitis, and cancer.

## Introduction: acid-base fluxes along the gastrointestinal tract

**Figure 1:**
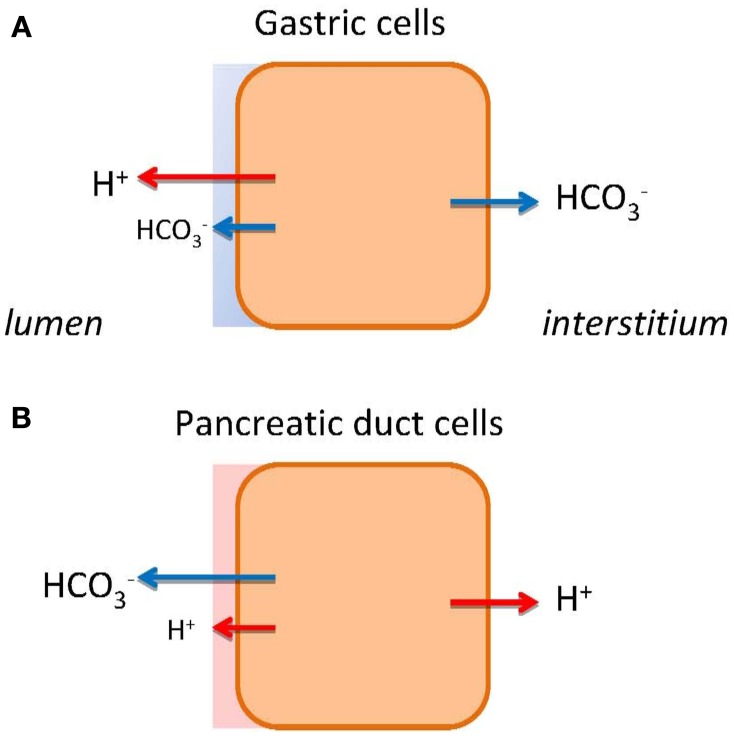
**HCO^−^_3_ and H^+^ transport in gastric cells (A) and pancreatic duct cells (B).** The models show schematically different types of epithelia as single cells. The transport of H^+^ or HCO^−^_3_ to the bulk luminal fluid is shown with large arrows. The small arrows on luminal side indicate HCO^−^_3_ and H^+^ secretions to the mucosal buffer zone. Flux of HCO^−^_3_ and H^+^ to the interstititum/blood side indicates expected alkaline or acid tides.

In multicellular organisms the digestive system exhibits marked acid/base segmentation and gradients across the epithelia. The most extreme examples of the acid/base transporters are the stomach and the pancreas, which conduct a vectorial transport of acid/base to one side and base/acid to the other side of the epithelium (Figure 1). In the stomach, the parietal cells of the pyloric glands secrete H^+^ toward lumen (HCl), leaving HCO^−^_3_ to be transported into the interstitium and blood. Thus, the phenomenon of the alkaline tide, i.e., higher blood pH in connection with digestion, is well known as part of the post-prandial gastric phase secretion, which in humans is relatively small compared to animals that ingest large amounts of food at one time (Rune and Lassen, 1968; Wang et al., 2001; Niv and Fraser, 2002). In the intestinal phase of digestion, pancreatic ducts secrete HCO^−^_3_-rich fluid that contributes to alkalinization of acid chyme in duodenum. The acid generated is then transported toward the interstitium, and one would expect an acid tide, depending on ingested food and passage through the stomach (Rune and Lassen, 1968; Ashley et al., 1994).

From these simple considerations several questions arise. Do the stomach and pancreas epithelia have some transport mechanisms in common, or do they solve the task of acid-base transport in different ways?

The molecular mechanism and regulation of stomach acid secretion is well established. In short, it involves gastric H^+^-K^+^-ATPases comprising of α1 and β subunits coded by *ATP4A* and *ATP4B* genes. These pumps are present in tubulovesicles of parietal cells and delivered to the luminal membranes in conjunction with specific K^+^ (KCNQ1, KCNJ15, KCNJ10) and Cl^−^ channels (CFTR, CLIC-6, SCL26A9), and thereby resulting in HCl secretion (Sachs et al., 2007; Forte and Zhu, 2010; Chu and Schubert, 2012). Gastric acid secretion is regulated by neural, hormonal, paracrine and chemical stimuli, e.g., acetylcholine, gastrin, ghrelin, histamine. As a protection against strong acid and pepsins, the surface epithelium secretes HCO^−^_3_, mucus and other factors, forming gastric diffusion barrier (Figure 1A). The validity of the model is confirmed by well-used drugs, including proton pump inhibitors and H_2_-histamine receptor blockers, to curb the peptic and duodenal ulcers and reflux diseases (Sachs et al., 2010). In contrast, we do not understand the mechanism behind pancreatic alkaline (HCO^−^_3_) secretion fully. Therefore, therapeutic intervention is not possible, e.g., for cystic fibrosis patients.

## Pancreatic secretion—contribution from acini and ducts

Pancreas is composed of two main types of epithelia—secretory acini and excretory ducts. Acini have relatively uniform morphology. They secrete digestive enzymes, NaCl-rich fluid and various factors that contribute to signaling in down-stream ducts. Studies on normal human and rodent pancreas, stimulated by predominantly acinar agonists, e.g., cholecytokinin (CCK), result in neutral or weakly alkaline pancreatic juice (Sewell and Young, 1975; You et al., 1983; Case and Argent, 1993). However, a recent study using acinar preparation and bioimaging techniques shows that acinar secretion is acidic due to acidic zymogen granules (ZG) (Behrendorff et al., 2010), although acidity of mature ZG has been discussed (Haanes and Novak, 2010; Chu and Schubert, 2012). Nevertheless, a potential acid load from acini challenging proximal ducts has been considered (Hegyi et al., 2011a). One possible defense mechanism could be activation of ducts by acinar agonist; generally this seems not to be the case. Alternatively, paracrine agonists such as ATP released by acini could stimulate ducts by purinergic signaling (Sørensen and Novak, 2001; Novak, 2008). Lastly, pancreatic ducts might have ability to sense and react to acid/base locally. There are a number of acid/base sensors at the single cell and whole organ level (Tresguerres et al., 2010; Brown and Wagner, 2012; DeCoursey, 2013). These include acid sensitive ASIC and TRP channels, HCO^−^_3_ sensitive adenylate cyclase, pH-sensitive K^+^ channels, and P2X receptors. Except for the latter two, which are expressed in pancreas (see below), other candidates remain to be explored.

**Figure 2:**
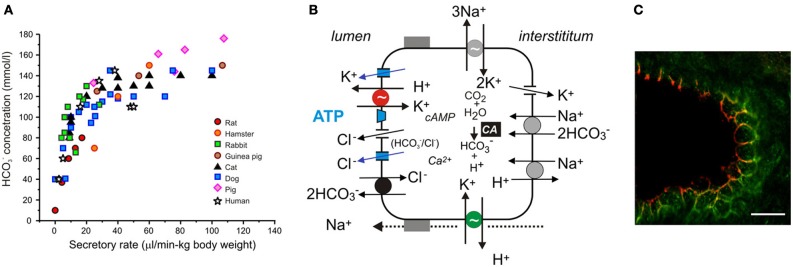
**Acid/base transport in pancreas. (A)** The relation between secretory rates and HCO^−^_3_ concentrations in pancreatic juice of various species. Secretions were stimulated by secretin and secretory rates were corrected for body weights. **(B)** The model of ion transport in a secreting pancreatic duct cell with novel transporters, channels and luminal purinergic signaling and receptors indicated in color and discussed in the review. Intracellular HCO^−^_3_ is derived from CO_2_ through the action of carbonic anhydrase (CA) and from HCO^−^_3_ uptake via the electrogenic Na^+^–HCO^−^_3_ cotransporter (pNBC, NBCe1). H^+^ is extruded at basolateral membrane by the Na^+^/H^+^ exchanger (NHE1). HCO^−^_3_ efflux across the luminal membrane is mediated by the electrogenic Cl^−^/HCO^−^_3_ exchanger (SLC26A6), and under certain conditions, through Cl^−^ channels. The luminal Cl^−^ channels are CFTR and TMEM16A (see text). There are a number of K^+^ channels expressed on the luminal and basolateral membranes, e.g., K_Ca_3.1, K_Ca_1.1, KCNQ1 (see text). The luminal and basolateral H^+^-K^+^-ATPases are indicated in red and green, and supposedly contribute to the luminal buffer zone and the H^+^ efflux to intersititum, respectively. Other ion channels and transporters, such as NHE3, SLC26A3, NBC3, NKCC1, and aquaporins have a differential distribution in the duct tree and for simplicity are not included in the model. **(C)** Immunolocalization of the gastric (red) and non-gastric (green) H^+^-K^+^ pumps in rat pancreatic duct. The bar is 20 μm. Modified from Novak et al., 2011.

Pancreatic ducts comprise 5–20% of the tissue mass, depending on the species; morphologically they are different - progressing from flat centroacinar cells, cuboidal cells in intercalated, and small intralobular ducts to columnar heterogenous cells lining larger distal ducts (Kodama, 1983; Ashizawa et al., 1997; Bouwens and Pipeleers, 1998). At large, it is accepted that pancreatic ducts secrete isotonic NaHCO_3_ rich fluid. However, the concentration of HCO^−^_3_ is not constant; it decreases with secretory rates—a pattern that is mirrored by Cl^−^. The HCO^−^_3_ excretory patterns are remarkably similar between various species, providing that secretory rates are corrected for the duct mass (Figure 2A). In early studies (Bro-Rasmussen et al., 1956), it was proposed that pancreatic secretion and ionic composition is a two stage process—primary secretion and ductal modification, the so called admixture hypothesis. Another, the exchange theory, also named the salvage mechanism, states that at lower secretory rates ductal transporters are presumably not saturated and therefore, are capable of exchanging luminal HCO^−^_3_ for interstitial Cl^−^. This exchange phenomenon was first demonstrated on the main cat duct (Case et al., 1969). The third explanation, regarding varying HCO^−^_3_ concentrations, pertains H^+^ secretion from acini (see above) or ducts (see below).

## Novel ion channels and pumps contributing to acid-base transport in pancreatic ducts

The ion transport models for pancreatic ducts have been described in several recent reviews (Steward et al., 2005; Steward and Ishiguro, 2009; Lee et al., 2012; Wilschanski and Novak, 2013). The outline of the model is given in Figure 2B. The following sections will focus on novel additions to the model.

### Proton pumps

Ion channels and transporters proposed in the classical model for HCO^−^_3_ secretion rely on gradients created by the Na^+^/K^+^-ATPase (Figure 2B). However, we cannot explain formation of high HCO^−^_3_ concentrations and the fact that inhibitors of NHE1, NBC (and NKCC1), and CA are relatively ineffective in blocking secretion (Grotmol et al., 1986; Fernandez-Salazar et al., 2004). One solution is that a primary pump could be involved, such as the vacuolar type H^+^-ATPase (V-H^+^-pump), to pump H^+^ out to interstitium and leave HCO^−^_3_ for the luminal transport. In one study, such vacuolar H^+^ pump on the basolateral membrane was proposed (Villanger et al., 1995) and detected immunohistochemically (Roussa et al., 2001). Several functional studies gave contradictory findings (Zhao et al., 1994; Ishiguro et al., 1996; de Ondarza and Hootman, 1997). Taking an inspiration from gastric glands, the colon and kidney distal tubules, we considered whether pancreatic ducts express H^+^-K^+^-ATPases. Indeed, we found that rodent ducts express both the gastric and non-gastric (colonic) types H^+^-K^+^-ATPases (Novak et al., 2011). Inhibition of these with proton pump inhibitors reduced pH_i_ recovery in response to acid loads; more importantly, they reduced secretion in isolated pancreatic ducts. Thus, these functional studies support the theory that pancreatic ducts resemble gastric glands—just working in reverse, expelling H^+^ toward the blood side and leaving HCO^−^_3_ for the luminal transport (Figure 1B). The immunohistochemical study showed that the H^+^-K^+^-ATPases (mainly colonic type) are localized to the basolateral membrane (Figure 2C).

However, H^+^-K^+^-ATPases, especially the gastric form, are also localized at or close to the luminal membrane (Figure 2C) (Novak et al., 2011). It seems counterintuitive to place H^+^ pumps on the HCO^−^_3_ secreting luminal membrane. Nevertheless, there are epithelia that are high HCO^−^_3_ secretors and yet express H^+^ pumps on the luminal membranes. For example, insect midgut and marine fish intestine have functional V-H^+^-ATPase on the luminal membranes (Wieczorek et al., 2009; Wood et al., 2010; Guffey et al., 2011). Also other epithelia, which are not high HCO^−^_3_ secretors (HCO^−^_3_ <25 mM), express various H^+^ pumps on the luminal membranes. For example, airway epithelia transport both base and acid, and the airway fluid layer is slightly acidic (Fischer and Widdicombe, 2006). Some studies provide evidence for the presence of bafilomycin A sensitive V-H^+^ pump (Inglis et al., 2003; Fischer and Widdicombe, 2006; Shan et al., 2012); other studies show that transport is sensitive to SCH28080, an inhibitor of gastric (and also non-gastric) H^+^-K^+^ pumps (Smith and Welsh, 1993; Poulsen and Machen, 1996). The non-gastric, ouabain-sensitive H^+^-K^+^-pumps were also demonstrated in some studies (Coakley et al., 2003; Krouse et al., 2004; Shan et al., 2012).

Coming back to the pancreatic luminal H^+^-K^+^ pumps, let us speculate what their function may be. They could help to defend the cell against intracellular acidification, although there is a redundancy of acid/base transporters including several NHEs, NBCs, and Cl^−^/HCO^−^_3_ exchangers (Figure 2B). Our proposal is—these luminal pumps are safeguarding luminal cell surface with acid secretions to protect against the bulk alkaline secretions, which at pH >8 would be caustic to cells. Thus, pancreatic ducts would have protective buffer (and mucus) zone, which is reminiscent to the buffer zone in the stomach, though achieved by H^+^ rather than HCO^−^_3_ secretion (Figures 1A,B). In addition, the luminal H^+^-K^+^ pumps would recirculate K^+^ extruded by the luminal K^+^ channels (Figure 2B). Lastly, luminal H^+^-K^+^ pumps in distal ducts would by virtue of H^+^ secretion have more impact on pancreatic juice composition at low flow rates and minor at high flow rates, thus, explaining excretory curves for HCO^−^_3_ (Figure 2A).

### Ca^2+^-activated Cl^−^ channels

In addition to CFTR-dependent secretion, a number of studies showed that agonists acting via Ca^2+^-signaling stimulate Ca^2+^-activated Cl^−^ channels (CaCC) and thus, could support duct secretion (Gray et al., 1989; Pahl and Novak, 1993; Winpenny et al., 1998; Szalmay et al., 2001; Pascua et al., 2009) (Figure 2B). The molecular identity of CaCC channels has been difficult to pinpoint [see (Duran et al., 2010)]. After suggestions of CCl-2 and bestrophins, the TMEM16/ANO family was discovered (Caputo et al., 2008; Schroeder et al., 2008; Yang et al., 2008), and especially TMEM16A/ANO1 became a CaCC favorite. Recent studies show that human duct cell lines express TMEM16A, which re-localizes from cytosol to the luminal membrane upon purinergic stimulation and gives rise to secretory potentials (Wang and Novak, 2013; Wang et al., 2013). In human pancreatic samples immunohistochemistry shows TMEM16A in centro-acinar and small ducts cells (Bergmann et al., 2011).

It is relevant to ask whether TMEM16A and/or Ca^2+^ signaling pathways lead to HCO^−^_3_ secretion. There are a few studies in support of this notion. For example, Ca^2+^ signaling via IRBIT stimulates NBCe1 (Shirakabe et al., 2006; Yang et al., 2009). A recent study on TMEM16A anion permeability shows that in HEK293 cell expression system and mouse salivary acinar cells the channel is directly modulated by calmodulin, which increases its HCO^−^_3_ permeability (Jung et al., 2013). This is supported by a study on *ex vivo* salivary glands stimulated with acetycholine, which induced production of HCO^−^_3_ rich pancreatic-like secretion when Cl^−^ transport was inhibited (Novak and Young, 1986). Nevertheless, it cannot be excluded that there are other molecular candidates for CaCC, or that CFTR can convey part of the Ca^2+^-activated Cl^−^ currents. The latter mechanism could involve Ca^2+^ sensitive adenylate cyclases and tyrosine kinases (Src2/Pyk complex), both of which could alter activity of CFTR, as shown for other epithelia (Billet and Hanrahan, 2013; Billet et al., 2013). Another effect at the CFTR level could be priming of some PKC isoforms that enhance CFTR activity [see (Billet and Hanrahan, 2013)]. Lastly, it is highly unlikely that Ca^2+^ mediated signaling stands alone, rather the two major signaling pathways of Ca^2+^ and cAMP/PKA act synergistically in pancreatic ducts, e.g., via IRBIT regulation of CFTR and SLC26A6 (Park et al., 2013).

### K^+^ channels

The driving force for Cl^−^ or HCO^−^_3_ exit is maintained by hyperpolarizing membrane potential created by opening of K^+^ channels, and G_K_ is both present on the basolateral and luminal membranes (Novak and Greger, 1988, 1991). Equivalent-circuit analysis has shown that stimulation of luminal K^+^ channels contributes with at least with 10% to the total conductance. Modeling in salivary glands confirms that such ratio of luminal to basolateral K^+^ channels would optimize secretion without destroying the transepithelial potential and transport (Cook and Young, 1989; Almassy et al., 2012). Furthermore, luminal K^+^ channels could contribute to secreted K^+^, as pancreatic juice contains 4–8 mM K^+^ (Sewell and Young, 1975; Caflisch et al., 1979; Seow et al., 1991). The molecular identity of some K^+^ channels in pancreatic ducts is known, however, the exact localization and function remains to be verified [see (Hayashi and Novak, 2013)]. The K_Ca_1.1 channels (maxi-K, BK, coded by *KCNMA1*) are present in pancreatic ducts (Hede et al., 2005; Venglovecz et al., 2011). The latter study proposes that these channels are expressed on the luminal membrane and activated by low concentrations of bile acids. However, earlier patch-clamp studies indicated that these channels were also located basolaterally (Gray et al., 1990; Hede et al., 1999). The K_Ca_3.1 channel (IK, SK4, coded by *KCNN4*) was demonstrated in pancreatic ducts (Hede et al., 2005; Jung et al., 2006; Hayashi et al., 2012). Immunolocalization indicates that K_Ca_3.1 is expressed on both membranes, though stronger on the luminal one (Figure 2B). Interestingly, the channel activator EBIO enhanced secretion potentials (Hayashi et al., 2012; Wang et al., 2013). Recent studies on pancreatic ducts offers molecular identities of several K^+^ channels, including KVLQT1, HERG, EAG2; Slick, and Slack (Hayashi et al., 2012), and interestingly the pH sensor TASK-2 (Fong et al., 2003). However, the function and regulation of these channels in pancreas physiology needs to be explored.

## Purinergic signaling

Pancreatic secretion regulated by hormonal and neural systems is well documented (Lee et al., 2012; Wilschanski and Novak, 2013). Paracrine regulation is less explored, but it is highly relevant as it allows regulation within the gland and integration of acinar and ductal responses. Pancreatic ducts can be regulated by acinar factors (trypsin, guanylin, ATP) as well as retrograde factors (bile acids) (Kulaksiz et al., 2001; Alvarez et al., 2004; Venglovecz et al., 2008; Pallagi et al., 2011; Wang and Novak, 2013). Here we concentrate on purinergic signaling and present evidence that this signaling could fine-tune and coordinate pancreatic secretion on several fronts. Pancreatic ducts express several types of purinergic receptors including members from the G-protein coupled receptor families (adenosine, P2Y) and ligand-gated ion channels (P2X receptor) families (Novak, 2008, 2011) that can potentially stimulate a variety of intracellular signaling pathways (Burnstock, 2007; Surprenant and North, 2009; Lenertz et al., 2011; Wiley et al., 2011; Bilbao et al., 2012). These receptors regulate pancreatic duct ion transport, mucin secretion, and survival of fibrogenic pancreatic stellate cells (Jung et al., 2004; Haanes et al., 2012). ATP originates from ZG where it is accumulated by the vesicular nucleotide transporter VNUT (Haanes and Novak, 2010), and in addition ATP is presumably released by nerves and ductal epithelium (Bodin and Burnstock, 2001; Novak, 2011; Burnstock and Novak, 2012). Various ecto-nucleotidases are expressed and secreted, and potentially ATP/ADP and adenosine are effective regulators of ductal functions (Sørensen et al., 2003; Kittel et al., 2004; Yegutkin et al., 2006; Burnstock and Novak, 2012).

ATP and UTP via P2 receptors have effects on intracellular Ca^2+^, intracellular pH, and transepithelial transport in both isolated ducts and *in vivo* pancreas (Ishiguro et al., 1999; Novak et al., 2010). The physiological response to nucleotides is side specific. Basolateral UTP inhibits secretion, most likely due to inhibition of K_Ca_1.1 channels, presumably to prevent overextension of ducts. In contrast, luminal UTP/ATP application causes duct secretion and activation and Cl^−^ and K^+^ channels (Hede et al., 1999; Ishiguro et al., 1999; Wang et al., 2013). In particular K_Ca_3.1 channel activation potentiates secretion (see above). It is well documented that purinergic receptor stimulation activates CFTR, Cl^−^/HCO^−^_3_ exchangers and TMEM16A on the luminal membrane (Namkung et al., 2003; Wang et al., 2013). Furthermore, P2 receptors activate CaCC and CFTR interdependently and synergistically, though exact receptors and signaling pathways remain to be elucidated (see above). In addition, some effects can be due to stimulation of A_2A_ and A_2B_ receptors, which stimulate CFTR (Novak et al., 2008).

A number of processes in purinergic signaling are pH sensitive, and this must be of relevance in pancreatic duct lumen. For example, nucleotidase activities, CD39 and CD73 types, are stimulated at alkaline pH 8–9 (Leal et al., 2005; Rucker et al., 2008), thus, favoring conversion of ATP to adenosine in duct lumen. Furthermore, purinergic receptors are also pH sensitive. From other preparations we know that extracellular acidification enhanced the potency of UTP up to 10 fold on the rat P2Y4 but not P2Y2 receptors (Wildman et al., 2003), and the P2X2 receptors was activated by acid pH (King et al., 1996). Extracellular alkalinization enhances activity the P2X4 and P2X7 receptors (Clarke et al., 2000; Liu et al., 2009). Several types of these receptors are expressed in duct lumen including the P2Y2 and P2X7 receptors, and these enhance pancreatic secretion and integrate acini-to-duct signaling (Novak, 2008; Novak et al., 2010).

## Summary and perspectives

The original cellular model for pancreatic HCO^−^_3_ secretion has been supplemented with molecular identities for many ion transporters/channels. The present review challenges present concepts by including active H^+^ pumps in the model, and by comparing basic processes in pancreas and stomach. Furthermore, we present new additions to the model—Ca^2+^-activated Cl^−^ and K^+^ channels, and propose that they work in synergy to regulate secretion. On the organ level, acini, and ducts integrate their function in acid/base transport and regulation, the latter exemplified by purinergic signaling. Further challenges lay in understanding dys-regulation of acid-base transport in pancreas pathophysiology. In CF patients and animal models, pancreatic juice pH decreases from values >8.1 to <6.6, and pancreas contributes to duodenal hyperacidity (Freedman et al., 2001; Uc et al., 2011) [see (Wilschanski and Novak, 2013)]. It is not clear whether the problem relates to ductal and/or acinar secretion. In acute pancreatitis, which has complex etiologies, it is now appreciated that defective pancreatic duct secretion can be the initiating factor (Lee and Muallem, 2008; Hegyi et al., 2011b). Finally, in several cancer types, various acid-base transporters and associated ion channels, such as NHE1, NBCn1, CAIX, TMEM16A, K_v_10.1, and K_Ca_3.1, change expression or function [see (Pedersen et al., 2013)]. Our knowledge about the role of acid-base transporters in pancreatic ductal adenocarcinoma clearly needs to be expanded, in order to provide potential diagnostic and therapeutic approaches.

### Conflict of interest statement

The authors declare that the research was conducted in the absence of any commercial or financial relationships that could be construed as a potential conflict of interest.

## Abbreviations

BK: big conductance K^+^ channel, also named K_Ca_1.1 and maxi-K^+^, coded by *KCNMA1*
CaCC: Ca^2+^-activated Cl^−^ channel, e.g., TMEM16A also known as ANO1
CA: carbonic anhydrase
CCK: cholecystokinin, CF, cystic fibrosis
[Ca^2+^]i: intracellular Ca^2+^ activity
CFTR: the cystic fibrosis transmembrane conductace regulator
EBIO: 1-ethyl-2-benzimidazolinone
G_K_: conductance for K^+^
H^+^-K^+^-ATPases or pumps: colonic type (coded by *ATP12A*) and gastric types (coded by *ATP4A* and *ATP4B*)
IK: intermediate conductance K^+^ channel, also named K_Ca_3.1
IRBIT: inositol 1,4,5-triphosphate (InsP3) receptor-binding protein released with InsP3
NBCe1 or pNBC: electrogenic Na^+^-HCO^−^_3_ transporter
NBCn1: electroneutral Na^+^-HCO_3_- transporter
NHE: Na^+^/H^+^ exchanger
NKCC1: Na^+^-K^+^-2Cl^−^ cotransporter
PKA: protein kinase A
PKC: proteins kinase C
SLC26A6: electrogenic Cl^−^-/2HCO^−^_3_- exchanger
VNUT: vesicular nucleotide transporter, SLC17A9
V-H^+^-pump: vacuolar type H^+^-ATPase
ZG: zymogen granules.

## References

[B1] Almassy J. Won J. H. Begenisich T. B. Yule D. I. (2012). Apical Ca^2+^-activated potassium channels in mouse parotid acinar cells. J. Gen. Physiol. 139, 121–133 10.1085/jgp.201110718 22291145 PMC3269790

[B2] Alvarez C. Regan J. P. Merianos D. Bass B. L. (2004). Protease-activated receptor-2 regulates bicarbonate secretion by pancreatic duct cells *in vitro*. Surgery 136, 669–676 10.1016/j.surg.2004.01.018 15349117

[B3] Ashizawa N. Endoh H. Hidaka K. Watanabe M. Fukumoto S. (1997). Three-dimensional structure of the rat pancreatic duct in normal and inflammated pancreas. Microsc. Res. Tech. 37, 543–556 10.1002/(SICI)1097-0029(19970601)37:5/6<543::AID-JEMT15>3.3.CO;2-Y 9220430

[B4] Ashley S. W. Schwarz M. Alvarez C. Nguyen T. N. Vdovenko A. Reber H. A. (1994). Pancreatic interstitial pH regulation: effects of secretory stimulation. Surgery 115, 503–509 8165542

[B5] Behrendorff N. Floetenmeyer M. Schwiening C. Thorn P. (2010). Protons released during pancreatic acinar cell secretion acidify the lumen and contribute to pancreatitis in mice. Gastroenterology 139, 1711-20, 1720.e1-5. 10.1053/j.gastro.2010.07.051 20691184

[B6] Bergmann F. Andrulis M. Hartwig W. Penzel R. Gaida M. M. Herpel E. (2011). Discovered on gastrointestinal stromal tumor 1 (DOG1) is expressed in pancreatic centroacinar cells and in solid-pseudopapillary neoplasms–novel evidence for a histogenetic relationship. Hum. Pathol. 42, 817–823 10.1016/j.humpath.2010.10.005 21295818

[B7] Bilbao P. S. Katz S. Boland R. (2012). Interaction of purinergic receptors with GPCRs, ion channels, tyrosine kinase and steroid hormone receptors orchestrates cell function. Purinergic Signal. 8, 91–103 10.1007/s11302-011-9260-9 21887492 PMC3286545

[B8] Billet A. Hanrahan J. W. (2013). The secret life of CFTR as a calcium-activated chloride channel. J. Physiol. 591, 5273–5278 10.1113/jphysiol.2013.261909 23959675 PMC3936366

[B9] Billet A. Luo Y. Balghi H. Hanrahan J. W. (2013). Role of tyrosine phosphorylation in the muscarinic activation of the Cystic Fibrosis Transmembrane Conductance Regulator (CFTR). J. Biol. Chem. 288, 21815–21823 10.1074/jbc.M113.479360 23760269 PMC3724638

[B10] Bodin P. Burnstock G. (2001). Purinergic signaling: ATP release. Neurochem. Res. 26, 959–969 10.1023/A:1012388618693 11699948

[B11] Bouwens L. Pipeleers D. G. (1998). Extra-insular beta cells associated with ductules are frequent in adult human pancreas. Diabetologia 41, 629–633 10.1007/s001250050960 9662042

[B12] Bro-Rasmussen F. Killmann S. A. Thaysen J. H. (1956). The composition of pancreatic juice as compared to sweat, parotid saliva and tears. Acta Physiol. Scand. 37, 97–113 10.1111/j.1748-1716.1956.tb01346.x 13361890

[B13] Brown D. Wagner C. A. (2012). Molecular mechanisms of acid-base sensing by the kidney. J. Am. Soc. Nephrol. 23, 774–780 10.1681/ASN.2012010029 22362904 PMC3338302

[B14] Burnstock G. (2007). Purine and pyrimidine receptors. Cell Mol. Life Sci. 64, 1471–1483 10.1007/s00018-007-6497-0 17375261 PMC11149472

[B15] Burnstock G. Novak I. (2012). Purinergic signaling in the pancreas in health and disease. J. Endocrinol. 213, 123–141 10.1530/JOE-11-0434 22396456

[B16] Caflisch C. R. Solomon S. Galey W. R. (1979). Exocrine ductal pCO_2_ in the rabbit pancreas. Pflugers Arch. 380, 121–125 10.1007/BF00582146 573439

[B17] Caputo A. Caci E. Ferrera L. Pedemonte N. Barsanti C. Sondo E. (2008). TMEM16A, a membrane protein associated with calcium-dependent chloride channel activity. Science 322, 590–594 10.1126/science.1163518 18772398

[B18] Case R. M. Argent B. E. (1993). Pancreatic duct cell secretion: control and mechanims of transport, in The Pancreas. Biology, Pathobiology, and Diseases, eds Go V. L. W. DiMagno E. P. Gardner J. D. Lebenthal E. Reber H. A. Scheele G. A. (New York, NY: Raven Press), 301–350

[B19] Case R. M. Harper A. A. Scratcherd T. (1969). The secretion of electrolytes and enzymes by the pancreas of the anaesthetized cat. J. Physiol. (Lond.) 201, 335–348 5780548 10.1113/jphysiol.1969.sp008759 PMC1351612

[B20] Chu S. Schubert M. L. (2012). Gastric secretion. Curr. Opin. Gastroenterol. 28, 587–593 10.1097/MOG.0b013e328358e5cc 22954692

[B21] Clarke C. E. Benham C. D. Bridges A. George A. R. Meadows H. J. (2000). Mutation of histidine 286 of the human P2X4 purinoceptor removes extracellular pH sensitivity. J. Physiol 523 (pt 3), 697–703 10.1111/j.1469-7793.2000.00697.x 10718748 PMC2269823

[B22] Coakley R. D. Grubb B. R. Paradiso A. M. Gatzy J. T. Johnson L. G. Kreda S. M. (2003). Abnormal surface liquid pH regulation by cultured cystic fibrosis bronchial epithelium. Proc. Natl. Acad. Sci. U.S.A. 100, 16083–16088 10.1073/pnas.2634339100 14668433 PMC307696

[B23] Cook D. I. Young J. A. (1989). Effect of K^+^ channels in the apical plasma membrane on epithelial secretion based on secondary active Cl^−^ transport. J. Membr. Biol. 110, 139–146 10.1007/BF01869469 2553974

[B24] DeCoursey T. E. (2013). Voltage-gated proton channels: molecular biology, physiology, and pathophysiology of the H(V) family. Physiol. Rev. 93, 599–652 10.1152/physrev.00011.2012 23589829 PMC3677779

[B25] de Ondarza J. Hootman S. R. (1997). Confocal microscopic analysis of intracellular pH regulation in isolated guinea pig pancreatic ducts. Am. J. Physiol. 272, G124–G134 9038885 10.1152/ajpgi.1997.272.1.G124

[B26] Duran C. Thompson C. H. Xiao Q. Hartzell H. C. (2010). Chloride channels: often enigmatic, rarely predictable. Annu. Rev. Physiol. 72, 95–121 10.1146/annurev-physiol-021909-135811 19827947 PMC2851227

[B27] Fernandez-Salazar M. P. Pascua P. Calvo J. J. Lopez M. A. Case R. M. Steward M. C. (2004). Basolateral anion transport mechanisms underlying fluid secretion by mouse, rat and guinea-pig pancreatic ducts. J. Physiol. (Lond.) 556, 415–428 10.1113/jphysiol.2004.061762 14978209 PMC1664956

[B28] Fischer H. Widdicombe J. H. (2006). Mechanisms of acid and base secretion by the airway epithelium. J. Membr. Biol. 211, 139–150 10.1007/s00232-006-0861-0 17091214 PMC2929530

[B29] Fong P. Argent B. E. Guggino W. B. Gray M. A. (2003). Characterization of vectorial chloride transport pathways in the human pancreatic duct adenocarcinoma cell line, HPAF. Am. J. Physiol. Cell Physiol. 285, C433–C445 10.1152/ajpcell.00509.2002 12711595

[B30] Forte J. G. Zhu L. (2010). Apical recycling of the gastric parietal cell H,K-ATPase. Annu. Rev. Physiol. 72, 273–296 10.1146/annurev-physiol-021909-135744 20148676

[B31] Freedman S. D. Kern H. F. Scheele G. A. (2001). Pancreatic acinar cell dysfunction in CFTR(-/-) mice is associated with impairments in luminal pH and endocytosis. Gastroenterology 121, 950–957 10.1053/gast.2001.27992 11606508

[B32] Gray M. A. Greenwell J. R. Garton A. J. Argent B. E. (1990). Regulation of maxi-K^+^ channels on pancreatic duct cells by cyclic AMP-dependent phosphorylation. J. Membr. Biol. 115, 203–215 10.1007/BF01868636 1695685

[B33] Gray M. A. Harris A. Coleman L. Greenwell J. R. Argent B. E. (1989). Two types of chloride channel on duct cells cultured from human fetal pancreas. Am. J. Physiol. 257, C240–C251 2475028 10.1152/ajpcell.1989.257.2.C240

[B34] Grotmol T. Buanes T. Bros O. Raeder M. G. (1986). Lack of effect of amiloride, furosemide, bumetanide and triamterene on pancreatic NaHCO_3_ secretion in pigs. Acta Physiol. Scand. 126, 593–600 10.1111/j.1748-1716.1986.tb07860.x 2424271

[B35] Guffey S. Esbaugh A. Grosell M. (2011). Regulation of apical H^+^-ATPase activity and intestinal HCO^−^_3_ secretion in marine fish osmoregulation. Am. J. Physiol. Regul. Integr. Comp. Physiol. 301, R1682–R1691 10.1152/ajpregu.00059.2011 21865541

[B36] Haanes K. A. Novak I. (2010). ATP storage and uptake by isolated pancreatic zymogen granules. Biochem. J. 429, 303–311 10.1042/BJ20091337 20441565

[B37] Haanes K. A. Schwab A. Novak I. (2012). The P2X7 receptor supports both life and death in fibrogenic pancreatic stellate cells. PLoS ONE 7:e51164 10.1371/journal.pone.0051164 23284663 PMC3524122

[B38] Hayashi M. Novak I. (2013). Molecular basis of potassium channels in pancreatic duct epithelial cells. Channels (Austin) 7, 1–10 10.4161/chan.26100 23962792 PMC4042478

[B39] Hayashi M. Wang J. Hede S. E. Novak I. (2012). An intermediate-conductance Ca^2+^-activated K^+^ channel is important for secretion in pancreatic duct cells. Am. J. Physiol. Cell Physiol. 303, C151–C159 10.1152/ajpcell.00089.2012 22555847

[B40] Hede S. E. Amstrup J. Christoffersen B. C. Novak I. (1999). Purinoceptors evoke different electrophysiological responses in pancreatic ducts. P2Y inhibits K^+^ conductance, and P2X stimulates cation conductance. J. Biol. Chem. 274, 31784–31791 10.1074/jbc.274.45.31784 10542200

[B41] Hede S. E. Amstrup J. Klaerke D. A. Novak I. (2005). P2Y2 and P2Y4 receptors regulate pancreatic Ca^2+^-activated K^+^ channels differently. Pflugers Arch. 450, 429–436 10.1007/s00424-005-1433-3 16075244

[B42] Hegyi P. Maleth J. Venglovecz V. Rakonczay Z. Jr. (2011a). Pancreatic ductal bicarbonate secretion: challenge of the acinar Acid load. Front. Physiol. 2:36 10.3389/fphys.2011.00036 21808623 PMC3139102

[B43] Hegyi P. Pandol S. Venglovecz V. Rakonczay Z. Jr. (2011b). The acinar-ductal tango in the pathogenesis of acute pancreatitis. Gut 60, 544–552 10.1136/gut.2010.218461 20876773 PMC6900977

[B44] Inglis S. K. Wilson S. M. Olver R. E. (2003). Secretion of acid and base equivalents by intact distal airways. Am. J. Physiol. Lung. Cell Mol. Physiol. 284, L855–L862 10.1152/ajplung.00348.2002 12676770

[B45] Ishiguro H. Naruse S. Kitagawa M. Hayakawa T. Case R. M. Steward M. C. (1999). Luminal ATP stimulates fluid and HCO^−^_3_ secretion in guinea-pig pancreatic duct. J. Physiol. (Lond) 519, 551–558 10.1111/j.1469-7793.1999.0551m.x 10457070 PMC2269526

[B46] Ishiguro H. Steward M. C. Wilson R. W. Case R. M. (1996). Bicarbonate secretion in interlobular ducts from guinea-pig pancreas. J. Physiol. (Lond.) 495 (pt 1), 179–191 8866361 10.1113/jphysiol.1996.sp021583 PMC1160734

[B47] Jung J. Nam J. H. Park H. W. Oh U. Yoon J. H. Lee M. G. (2013). Dynamic modulation of ANO1/TMEM16A. Proc. Natl. Acad. Sci. U.S.A. 110, 360–365 10.1073/pnas.1211594110 23248295 PMC3538232

[B48] Jung S. R. Kim K. Hille B. Nguyen T. D. Koh D. S. (2006). Pattern of Ca^2+^ increase determines the type of secretory mechanism activated in dog pancreatic duct epithelial cells. J. Physiol. 576, 163–178 10.1113/jphysiol.2006.114876 16857709 PMC1995640

[B49] Jung S. R. Kim M. H. Hille B. Nguyen T. D. Koh D. S. (2004). Regulation of exocytosis by purinergic receptors in pancreatic duct epithelial cells. Am. J. Physiol. Cell Physiol. 286, C573–C579 10.1152/ajpcell.00350.2003 14602582

[B50] King B. F. Ziganshina L. E. Pintor J. Burnstock G. (1996). Full sensitivity of P2X2 purinoceptor to ATP revealed by changing extracellular pH. Br. J. Pharmacol. 117, 1371–1373 10.1111/j.1476-5381.1996.tb15293.x 8730726 PMC1909447

[B51] Kittel A. Pelletier J. Bigonnesse F. Guckelberger O. Kordas K. Braun N. (2004). Localization of Nucleoside Triphosphate Diphosphohydrolase-1 (NTPDase1) and NTPDase2 in Pancreas and Salivary Gland. J. Histochem. Cytochem. 52, 861–871 10.1369/jhc.3A6167.2004 15208353

[B52] Kodama T. (1983). A light and electron microscopic study on the pancreatic ductal system. Acta Pathol. Jpn. 33, 297–321 6346783 10.1111/j.1440-1827.1983.tb01419.x

[B53] Krouse M. E. Talbott J. F. Lee M. M. Joo N. S. Wine J. J. (2004). Acid and base secretion in the Calu-3 model of human serous cells. Am. J. Physiol. Lung. Cell Mol. Physiol. 287, L1274–L1283 10.1152/ajplung.00036.2004 15310554

[B54] Kulaksiz H. Schmid A. Honscheid M. Eissele R. Klempnauer J. Cetin Y. (2001). Guanylin in the human pancreas: a novel luminocrine regulatory pathway of electrolyte secretion via cGMP and CFTR in the ductal system. Histochem. Cell Biol. 115, 131–145 10.1007/s004180000244 11444148

[B55] Leal D. B. Streher C. A. Neu T. N. Bittencourt F. P. Leal C. A. da Silva J. E. (2005). Characterization of NTPDase (NTPDase1; ecto-apyrase; ecto-diphosphohydrolase; CD39; EC 3.6.1.5) activity in human lymphocytes. Biochim. Biophys. Acta 1721, 9–15 10.1016/j.bbagen.2004.09.006 15652174

[B56] Lee M. G. Muallem S. (2008). Pancreatitis: the neglected duct. Gut 57, 1037–1039 10.1136/gut.2008.150961 18628371

[B57] Lee M. G. Ohana E. Park H. W. Yang D. Muallem S. (2012). Molecular mechanism of pancreatic and salivary gland fluid and HCO^−^_3_ secretion. Physiol. Rev. 92, 39–74 10.1152/physrev.00011.2011 22298651 PMC3667394

[B58] Lenertz L. Y. Gavala M. L. Zhu Y. Bertics P. J. (2011). Transcriptional control mechanisms associated with the nucleotide receptor P2X7, a critical regulator of immunologic, osteogenic, and neurologic functions. Immunol. Res. 50, 22–38 10.1007/s12026-011-8203-4 21298493 PMC3203638

[B59] Liu X. Ma W. Surprenant A. Jiang L. H. (2009). Identification of the amino acid residues in the extracellular domain of rat P2X(7) receptor involved in functional inhibition by acidic pH. Br. J. Pharmacol. 156, 135–142 10.1111/j.1476-5381.2008.00002.x 19068080 PMC2697781

[B60] Namkung W. Lee J. A. Ahn W. Han W. Kwon S. W. Ahn D. S. (2003). Ca^2+^ activates cystic fibrosis transmembrane conductance regulator- and Cl^−^ -dependent HCO_3_ transport in pancreatic duct cells. J. Biol. Chem. 278, 200–207 10.1074/jbc.M207199200 12409301

[B61] Niv Y. Fraser G. M. (2002). The alkaline tide phenomenon. J. Clin. Gastroenterol. 35, 5–8 10.1097/00004836-200207000-00003 12080218

[B62] Novak I. (2008). Purinergic receptors in the endocrine and exocrine pancreas. Purinergic Signal. 4, 237–253 10.1007/s11302-007-9087-6 18368520 PMC2486345

[B63] Novak I. (2011). Purinergic signaling in epithelial ion transport—regulation of secretion and absorption. Acta Physiologica 202, 501–522 10.1111/j.1748-1716.2010.02225.x 21073662

[B64] Novak I. Greger R. (1988). Electrophysiological study of transport systems in isolated perfused pancreatic ducts: properties of the basolateral membrane. Pflügers Arch. 411, 58–68 10.1007/BF00581647 3353213

[B65] Novak I. Greger R. (1991). Effect of bicarbonate on potassium conductance of isolated perfused rat pancreatic ducts. Pflügers Arch. 419, 76–83 10.1007/BF00373750 1945765

[B66] Novak I. Hede S. E. Hansen M. R. (2008). Adenosine receptors in rat and human pancreatic ducts stimulate chloride transport. Pflugers Arch. 456, 437–447 10.1007/s00424-007-0403-3 18057956

[B67] Novak I. Jans I. M. Wohlfahrt L. (2010). Effect of P2X7 receptor knockout on exocrine secretion of pancreas, salivary glands and lacrimal glands. J. Physiol. (Lond) 588 (pt 18), 3615–3627 10.1113/jphysiol.2010.190017 20643770 PMC2988522

[B68] Novak I. Wang J. Henriksen K. L. Haanes K. A. Krabbe S. Nitschke R. (2011). Pancreatic bicarbonate secretion involves two proton pumps. J. Biol. Chem. 286, 280–289 10.1074/jbc.M110.136382 20978133 PMC3012985

[B69] Novak I. Young J. A. (1986). Two independent anion transport systems in rabbit mandibular salivary glands. Pflugers Arch. 407, 649–656 10.1007/BF00582647 3797220

[B70] Pahl C. Novak I. (1993). Effect of vasoactive intestinal peptide, carbachol and other agonists on cell membrane voltage of pancreatic duct cells. Pflügers Arch. 424, 315–320 10.1007/BF00384358 8414920

[B71] Pallagi P. Venglovecz V. Rakonczay Z. Jr. Borka K. Korompay A. Ozsvari B. (2011). Trypsin reduces pancreatic ductal bicarbonate secretion by inhibiting CFTR Cl channels and luminal anion exchangers. Gastroenterology 141, 2228–2239 10.1053/j.gastro.2011.08.039 21893120 PMC3273991

[B72] Park S. Shcheynikov N. Hong J. H. Zheng C. Suh S. H. Kawaai K. (2013). Irbit mediates synergy between Ca^2+^ and cAMP signaling pathways during epithelial transport in mice. Gastroenterology 145, 232–241 10.1053/j.gastro.2013.03.047 23542070 PMC3696401

[B73] Pascua P. Garcia M. Fernandez-Salazar M. P. Hernandez-Lorenzo M. P. Calvo J. J. Colledge W. H. (2009). Ducts isolated from the pancreas of CFTR-null mice secrete fluid. Pflugers Arch. 459, 203–214 10.1007/s00424-009-0704-9 19655163

[B74] Pedersen S. F. Hoffmann E. K. Novak I. (2013). Cell volume regulation in epithelial physiology and cancer. Front. Physiol. 4, 233 10.3389/fphys.2013.00233 24009588 PMC3757443

[B75] Poulsen J. H. Machen T. E. (1996). HCO_3_-dependent pHi regulation in tracheal epithelial cells. Pflugers Arch. 432, 546–554 10.1007/s004240050168 8766016

[B76] Roussa E. Alper S. L. Thevenod F. (2001). Immunolocalization of anion exchanger AE2, Na^+^/H^+^ exchangers NHE1 and NHE4, and vacuolar type H^+^-ATPase in rat pancreas. J. Histochem. Cytochem. 49, 463–474 10.1177/002215540104900406 11259449

[B77] Rucker B. Almeida M. E. Libermann T. A. Zerbini L. F. Wink M. R. Sarkis J. J. (2008). E-NTPDases and ecto-5'-nucleotidase expression profile in rat heart left ventricle and the extracellular nucleotide hydrolysis by their nerve terminal endings. Life Sci. 82, 477–486 10.1016/j.lfs.2007.12.003 18201730

[B78] Rune S. J. Lassen N. A. (1968). Diurnal variations in the acid-base balance of blood. Scand. J. Clin. Lab. Invest. 22, 151–156 10.3109/00365516809160961 5755924

[B79] Sachs G. Shin J. M. Hunt R. (2010). Novel approaches to inhibition of gastric acid secretion. Curr. Gastroenterol. Rep. 12, 437–447 10.1007/s11894-010-0149-5 20924727 PMC2974194

[B80] Sachs G. Shin J. M. Vagin O. Lambrecht N. Yakubov I. Munson K. (2007). The gastric H,K ATPase as a drug target: past, present, and future. J. Clin. Gastroenterol. 41 (Suppl. 2), S226–S242 10.1097/MCG.0b013e31803233b7 17575528 PMC2860960

[B81] Schroeder B. C. Cheng T. Jan Y. N. Jan L. Y. (2008). Expression cloning of TMEM16A as a calcium-activated chloride channel subunit. Cell 134, 1019–1029 10.1016/j.cell.2008.09.003 18805094 PMC2651354

[B82] Seow K. T. F. P. Case R. M. Young J. A. (1991). Pancreatic secretion by the anaesthetized rabbit in response to secretin, cholecystokinin, and carbachol. Pancreas 6, 385–391 10.1097/00006676-199107000-00002 1876596

[B83] Sewell W. A. Young J. A. (1975). Secretion of electrolytes by the pancreas of the anaesthetized rat. J. Physiol. (Lond.) 252, 379–396 1206529 10.1113/jphysiol.1975.sp011149 PMC1348450

[B84] Shan J. Liao J. Huang J. Robert R. Palmer M. L. Fahrenkrug S. C. (2012). Bicarbonate-dependent chloride transport drives fluid secretion by the human airway epithelial cell line Calu-3. J. Physiol. 590, 5273–5297 10.1113/jphysiol.2012.236893 22777674 PMC3515819

[B85] Shirakabe K. Priori G. Yamada H. Ando H. Horita S. Fujita T. (2006). IRBIT, an inositol 1,4,5-trisphosphate receptor-binding protein, specifically binds to and activates pancreas-type Na^+^/HCO^−^_3_ cotransporter 1 (pNBC1). Proc. Natl. Acad. Sci. U.S.A. 103, 9542–9547 10.1073/pnas.0602250103 16769890 PMC1480443

[B86] Smith J. J. Welsh M. J. (1993). Fluid and electrolyte transport by cultured human airway epithelia. J. Clin. Invest. 91, 1590–1597 10.1172/JCI116365 8473502 PMC288135

[B87] Sørensen C. E. Amstrup J. Rasmussen H. N. Ankorina-Stark I. Novak I. (2003). Rat pancreas secretes particulate ecto-nucleotidase CD39.J. Physiol. (Lond.) 551, 881–892 10.1113/jphysiol.2003.049411 12832497 PMC2343304

[B88] Sørensen C. E. Novak I. (2001). Visualization of ATP release in pancreatic acini in response to cholinergic stimulus. Use of fluorescent probes and confocal microscopy. J. Biol. Chem. 276, 32925–32932 10.1074/jbc.M103313200 11387334

[B89] Steward M. C. Ishiguro H. (2009). Molecular and cellular regulation of pancreatic duct cell function. Curr. Opin. Gastroenterol. 25, 447–453 10.1097/MOG.0b013e32832e06ce 19571747

[B90] Steward M. C. Ishiguro H. Case R. M. (2005). Mechanisms of bicarbonate secretion in the pancreatic duct. Annu. Rev. Physiol. 67, 377–409 10.1146/annurev.physiol.67.031103.153247 15709963

[B91] Surprenant A. North R. A. (2009). Signaling at purinergic P2X receptors. Annu. Rev. Physiol. 71, 333–359 10.1146/annurev.physiol.70.113006.100630 18851707

[B92] Szalmay G. Varga G. Kajiyama F. Yang X. S. Lang T. F. Case R. M. (2001). Bicarbonate and fluid secretion evoked by cholecystokinin, bombesin and acetylcholine in isolated guinea-pig pancreatic ducts. J. Physiol. (Lond.) 535, 795–807 10.1111/j.1469-7793.2001.00795.x 11559776 PMC2278811

[B93] Tresguerres M. Buck J. Levin L. R. (2010). Physiological carbon dioxide, bicarbonate, and pH sensing. Pflugers Arch. 460, 953–964 10.1007/s00424-010-0865-6 20683624 PMC2967379

[B94] Uc A. Stoltz D. A. Ludwig P. Pezzulo A. Griffin M. bu-El-Haija M. (2011). Pancreatic and biliary secretion differ in cystic fibrosis and wild-type pigs. J. Cystic Fibrosis 10, S69 10.1016/S1569-1993(11)60285-3 22936270

[B95] Venglovecz V. Hegyi P. Rakonczay Z. Jr. Tiszlavicz L. Nardi A. Grunnet M. (2011). Pathophysiological relevance of apical large-conductance Ca^2+^-activated potassium channels in pancreatic duct epithelial cells. Gut 60, 361–369 10.1136/gut.2010.214213 20940280

[B96] Venglovecz V. Rakonczay Z. Jr. Ozsvari B. Takacs T. Lonovics J. Varro A. (2008). Effects of bile acids on pancreatic ductal bicarbonate secretion in guinea pig. Gut 57, 1102–1112 10.1136/gut.2007.134361 18303091

[B97] Villanger O. Veel T. Raeder M. G. (1995). Secretin causes H^+^/HCO^−^_3_ secretion from pig pancreatic ductules by vacuolar-type H^+^-adenosine triphosphatase. Gastroenterology 108, 850–859 10.1016/0016-5085(95)90460-3 7875488

[B98] Wang J. Haanes K. A. Novak I. (2013). Purinergic regulation of CFTR and Ca^2+^-activated Cl^−^ channels and K^+^ channels in human pancreatic duct epithelium. Am. J. Physiol. Cell Physiol. 304, C673–C684 10.1152/ajpcell.00196.2012 23364268

[B99] Wang J. Novak I. (2013). Ion transport in human pancreatic duct epithelium, Capan-1 cells, is regulated by secretin, VIP, acetylcholine, and purinergic receptors. Pancreas 42, 452–460 10.1097/MPA.0b013e318264c302 22982819

[B100] Wang T. Busk M. Overgaard J. (2001). The respiratory consequences of feeding in amphibians and reptiles. Comp. Biochem. Physiol. A Mol. Integr. Physiol. 128, 535–549 10.1016/S1095-6433(00)00334-2 11246043

[B101] Wieczorek H. Beyenbach K. W. Huss M. Vitavska O. (2009). Vacuolar-type proton pumps in insect epithelia. J. Exp. Biol. 212, 1611–1619 10.1242/jeb.030007 19448071 PMC2683008

[B102] Wildman S. S. Unwin R. J. King B. F. (2003). Extended pharmacological profiles of rat P2Y2 and rat P2Y4 receptors and their sensitivity to extracellular H^+^ and Zn^2+^ ions. Br. J. Pharmacol. 140, 1177–1186 10.1038/sj.bjp.0705544 14581177 PMC1574132

[B103] Wiley J. S. Sluyter R. Gu B. J. Stokes L. Fuller S. J. (2011). The human P2X7 receptor and its role in innate immunity. Tissue Antigens 78, 321–332 10.1111/j.1399-0039.2011.01780.x 21988719

[B104] Wilschanski M. Novak I. (2013). The cystic fibrosis of exocrine pancreas. Cold Spring Harb. Perspect. Med. 3, a009746 10.1101/cshperspect.a009746 23637307 PMC3633181

[B105] Winpenny J. P. Harris A. Hollingsworth M. A. Argent B. E. Gray M. A. (1998). Calcium-activated chloride conductance in a pancreatic adenocarcinoma cell line of ductal origin (HPAF) and in freshly isolated human pancreatic duct cells. Pflugers Arch. 435, 796–803 10.1007/s004240050586 9518508

[B106] Wood C. M. Bucking C. Grosell M. (2010). Acid-base responses to feeding and intestinal Cl^−^ uptake in freshwater- and seawater-acclimated killifish, Fundulus heteroclitus, an agastric euryhaline teleost. J. Exp. Biol. 213, 2681–2692 10.1242/jeb.039164 20639430

[B107] Yang D. Shcheynikov N. Zeng W. Ohana E. So I. Ando H. (2009). IRBIT coordinates epithelial fluid and HCO^−^_3_ secretion by stimulating the transporters pNBC1 and CFTR in the murine pancreatic duct. J. Clin. Invest. 119, 193–202 10.1172/JCI36983 19033647 PMC2613461

[B108] Yang Y. D. Cho H. Koo J. Y. Tak M. H. Cho Y. Shim W. S. (2008). TMEM16A confers receptor-activated calcium-dependent chloride conductance. Nature 455, 1210–1215 10.1038/nature07313 18724360

[B109] Yegutkin G. G. Samburski S. S. Jalkalen S. Novak I. (2006). ATP-consuming and ATP-generating enzymes secreted by pancreas. J. Biol. Chem. 281, 29441–29447 10.1074/jbc.M602480200 16885159

[B110] You C. H. Rominger J. M. Chey W. Y. (1983). Potentiation effect of cholecystokinin-octapeptide on pancreatic bicarbonate secretion stimulated by a physiologic dose of secretin in humans. Gastroenterology 85, 40–45 6303892

[B111] Zhao H. Star R. A. Muallem S. (1994). Membrane localization of H^+^ and HCO^−^_3_ transporters in the rat pancreatic ducts. J. Gen. Physiol. 104, 57–85 10.1085/jgp.104.1.57 7964596 PMC2229195

